# A Brief for Military Education in School and Colleges

**Published:** 1915-10

**Authors:** 


**A Brief For Military Education in School and Colleges.** Lucien Howe, Buffalo, Jour. of the Military Service Institution, July-August. Without advocating militarism, Dr. Howe appreciates the value of a trained militia, in the original sense but still more so, the physical and moral stamina derived from military training. 56 per cent. of applicants for enlistment in the U. S. Army in 1912 were rejected for physical causes and of 1256 students at the University of Pennsylvania, only 97 were considered physically perfect. Gymnastic and postural training alone is of great value and should be made interesting, a matter of pride and might even be rewarded by government prizes. 400,000 boys leave the grammar schools every year. Either for civil life or in a military emergency, the results of physical training would soon be conspicuous.

